# Study on TCM Tongue Image Segmentation Model Based on Convolutional Neural Network Fused with Superpixel

**DOI:** 10.1155/2022/3943920

**Published:** 2022-03-08

**Authors:** Han Zhang, Rongrong Jiang, Tao Yang, Jiayi Gao, Yi Wang, Junfeng Zhang

**Affiliations:** ^1^School of Artificial Intelligence and Information Technology, Nanjing University of Chinese Medicine, Nanjing, China; ^2^School of Nursing, Nanjing University of Chinese Medicine, Nanjing, China; ^3^School of Information Management, Nanjing University, Nanjing, China; ^4^School of Medicine & Holistic Integrative Medicine, Nanjing University of Chinese Medicine, Nanjing, China

## Abstract

Tongue image segmentation is a base work of TCM tongue processing. Nowadays, deep learning methods are widely used on tongue segmentation, which has better performance than conventional methods. However, when the tongue color is close to the color of the adjoining area, the contour of tongue segmentation by deep learning may be coarse which could influence the subsequent analysis. Here a novel tongue image segmentation model based on a convolutional neural network fused with superpixel was proposed to solve the problem. *Methods*. On the basis of a convolutional neural network fused with superpixel, the novel tongue image segmentation model SpurNet was proposed in this study. The residual structure of ResNet18 was introduced as the feature extraction layer on the encoding path, to construct the first stage processing module UrNet of SpurNet. The superpixel segmentation was fused with UrNet to form the second stage process of SpurNet. To verify the effect of SpurNet. The models before and after fusion with superpixel, classical image segmentation models FCN and DeepLab were compared with SpurNet on the dataset of 367 manually labeled tongue images. *Results*. The SpurNet model performance test with 10-fold cross-validation showed PA of 0.9145 ± 0.0043, MPA of 0.9168 ± 0.0048, MIoU of 0.8417 ± 0.0072 and FWIoU of 0.8454 ± 0.0072. Relative to FCN, DeepLab and their superpixel fused models, the SpurNet model was superior in tongue image segmentation and could increase PA by 1.91%–3.17%, MPA by 1.38%–2.61%, MIoU by 3.09%–5.07%, and FWIoU by 3.11%–5.08%. Compared to UrNet, the first stage processing module, the SpurNet model also increased the PA, MPA, MIoU and FWIoU by 0.15%, 0.09%, 0.24% and 0.24%, respectively. *Conclusion*. The SpurNet model, after fusing with superpixel image segmentation, can better accomplish the task of tongue image segmentation, more accurately process the margins of tongue and resolve the over-segmentation and under-segmentation. The thought of this study is a new exploration in the field of tongue image segmentation, which could provide a reference for the modern research on TCM tongue images.

## 1. Introduction

Traditional Chinese medicine (TCM) is a treasure of ancient science and the intelligence of Chinese culture that has been celebrated in China for thousands of years. Its effective practical methods have made noble contributions to the prosperity and healthy development of the Chinese nation. There are four basic diagnostic methods in TCM, including inspection, auscultation and olfaction, inquisition and pulse diagnosis, in which inspection is the most effective and visual one. As an old saying goes, “there may be a false pulse but no false tongue feature.” The internal organs in human body are connected with the tongue through meridians and collaterals, and the patients' body constitutions can be preliminarily diagnosed, and the medical conditions can be analyzed by the clinical physicians via observation of the tongue features, including tongue color, tongue shape and tongue state. This noninvasive diagnostic method enables tongue diagnosis as a necessary step in the TCM diagnosis [1]. However, since the results of tongue diagnosis only depend on the accumulated experience of the clinical physicians, cannot be easily replicated, and may be affected by the exogenous factors, objectification and standardization of tongue feature diagnosis has become a hot topic in the studies of TCM informatics. Tongue image segmentation [2–4], as the first step of intellectualization of tongue features, has provided a basis for the subsequent tongue processing and analysis, and has become an inseparable part in the process of intellectualizing tongue diagnosis [5,6].

Traditional tongue image processing mainly depends on conventional image processing techniques [7–9]. The tongue segmentation, also known as the pixel-level classification task, is to remove the regions (including lips, tooth and the space between lips and teeth) unrelated to the tongue based on the unprocessed tongue image, similar to the foreground and background classification for each pixel in the tongue image. The common traditional segmentation algorithms include threshold segmentation [10–12], gradient segmentation [4, 13] and GrabCut [14–16]. For example, Ren JJ [17] proposed a color tongue image auto-thresholding segmentation algorithm based on RGB spatial histogram and used this threshold for the tongue image segmentation in the gray space. Zhang *L* and others. [18] proposed a segmentation method based on gray projection and threshold-adaptive method. Fu ZC and others. [19] adopted radial edge detection to obtain the approximate contour of tongue, and then used paired color removal method to remove the lips, and applied snake model to obtain the precise contour of tongue finally. Shi MJ and others. [20] combined geometric snake model and the parameterized GVFSnake model and proposed a novel tongue auto-segmentation method. Nonetheless, above methods have certain limitations: As to auto-adaptive threshold segmentation method, the color of the regions (such as lips) outside of tongue are similar to the color of tongue, and single threshold cannot accurately distinguish the foreground and background; Snake algorithm easily runs into local extremum and neglect some fine characteristics during the process of contour energization; Grabcut is related to the prior knowledge and maximally depends on the range of interest (ROI) given by the user with a degree of automation when segmentation the tongue features.

With the development of deep learning, some scholars have attempted to use deep learning algorithms into tongue feature segmentation [21–23]. Wang *L* and others. [24] proposed a segmentation method of tongue based on the two-phase convolutional neural network. Zhang *X* [25] used atrous spatial pyramid pooling (ASPP) module to perceive the multiresolution characteristics of the tongue features, and then combined the deep convolutional neural network with the full connection conditional random field to refine the margins of tongue image. Jiang *L* and others. [26] adopted convolutional neural network of the enhanced HSV (hue, saturation, value) color model into tongue segmentation. Lin B and others. [27] proposed DeepLingue, an end-to-end depth convolutional neural network model based on ResNet and discovered that this model was not affected by light or the size of tongue image, and was superior to conventional segmentation algorithms in the segmentation velocity ratio and accuracy. Li et al. [28] designed an iterative cross-domain tongue segmentation framework based on UNet and transfer learning. Gholami et al. [29] used to separate the tongue region from the face image using R–CNN to provide images for subsequent tongue classification.

It has been known from above studies that deep learning methods are widely used on tongue segmentation. However, when the tongue color is close to the color of the adjoining area, the contour of tongue segmentation may be coarse which could lead negative influence on the subsequent quantitative analysis. Therefore this study designed a TCM tongue segmentation algorithm by fusing the convolutional neural network with the superpixels to achieve more accurate processing of the margins of tongue and resolve the over-segmentation and under-segmentation issues. To perform stable segmentation and refine the margins of tongue in different photography environment, this study mainly completed following tasks:

1. Designed a novel tongue segmentation model which introduced ResNet18 residual structure as the characteristics abstraction layer of coding path based on UNet.
2. Proposed to use superpixel image segmentation to optimize and increase the segmentation accuracy in view of the noisiness of the tongue image background and certain errors between the convolutional neural network in processing the margins of tongue.
3. Compared the segmentation effectiveness of the model before and after fusion with superpixels, and compared the novel model with the classic convolutional networks (FCN and Deeplab).

The remainder of the paper was organized as follows: The materials and methods were specified in section 2, the results of the established model was introduced, analyzed and discussed in section 3 and section 4, and the study was summarized in section 5.

## 2. Materials and Methods

### 2.1. Sampling and Labeling of the Tongue Images

The experimental data were acquired by the tongue diagnosis study group from the Nanjing University of Chinese Medicine. The study group collected the tongue images of 257 patients with gastric carcinoma using smartphones during the treatment in hospital, and finally selected 367 tongue images after excluding the images that did not contain the tongue regions (multiple tongue images were collected from the same patients at different visit date) to establish the tongue image dataset. Compared with traditional tongue image acquisition equipment, it is more convenient and efficient to use smartphones to collect tongue images. Moreover, models built on common tongue images collected by smartphones have more applicability and operability, which can be embedded in apps and provide AI services of tongue processing.

**Figure 1:**
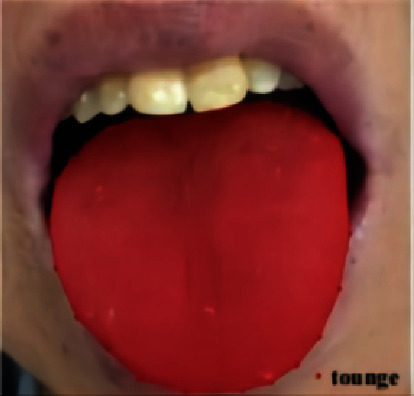
Labeling result of a tongue image.

The tongue contour was labeled using the graphic interface provided by the Python Labelme package. The tongue contour was drawn after the name of the region category was set (Figure 1). The labeled tongue feature data were used to establish the sample set for tongue feature segmentation.

### 2.2. Framework of Tongue Segmentation Model Based on Convolutional Neural Network Fused with Superpixel

**Figure 2:**
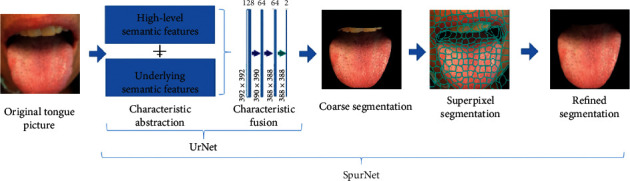
SpurNet framework.

As to tongue images, most of them had ill-defined margins and needed to refer to more low-resolution information and use context fusion to achieve precise segmentation. Moreover, since the tongue structure was relatively fixed and the semantics of segmentation target was definite and straightforward, and high-resolution characteristics could provide more location information. Therefore, SpurNet, a TCM tongue feature segmentation model, was proposed, with structure specified in Figure 2.

To extract the underlying semantic characteristics and high-level semantic characteristics of tongue images, UNet [30] was used as the model skeleton, and ResNet18 [31] residual network was introduced as the feature extraction layer of UNet coding path to improve the sensitivity of feature mapping to output changes and improve tongue segmentation accuracy, A tongue image segmentation model UrNet (UNet-Resnet18) containing encoding and decoding is constructed to realize rough segmentation of tongue. On this basis, superpixel characteristics were added for the optimizing process of the coarse segmentation results. It was verified by subsequent experiments that SpurNet (UrNet + superpixel) could effectively resolve the issues including unsmoothness of margin segmentation as well as the over-segmentation and under-segmentation of the background.

**Figure 3:**
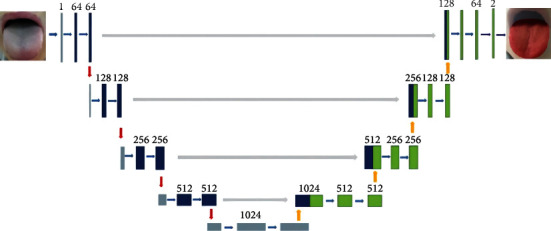
UNet structure.

The UNet is the base of SpurNet. In the classic UNet network structure, an end-to-end “U”-shaped encoder-decoder framework was used, as shown in Figure 3. It contained 3 parts: Extraction of the features of backbone, a fusion of characteristics and tongue segmentation. In the extraction of the features of backbone, skip connection was performed for high-level semantic features and underlying semantic features to ensure that the downstream feature maps could fuse more low-dimension features (most of which were marginal information), so that multi-scale features could be efficiently fused.

**Figure 4:**
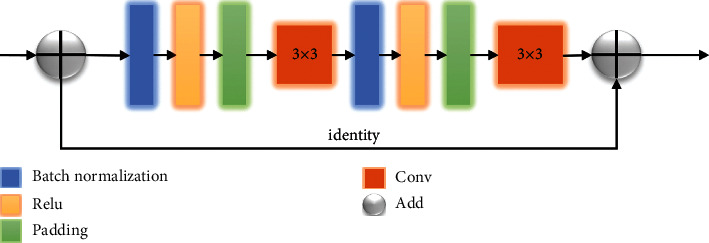
One unit in UrNet.

Since the backbone feature extraction of UNet was composed of common CBR modules (Conv + BN + ReLU), this study learned the stronger Resnet as the BackBone of UNet. Residual links were introduced into the original UNet, and the encoder was connected to the decoder, so as to retain the lost information in different layers in the encoding part, enhance the perception of feature mapping to the changes of output, and promote the accuracy of tongue segmentation. The structure of one Unit in UrNet is shown in Figure 4.

On the basis of UrNet, the SpurNet proposed in this study further considered the over-segmentation and under-segmentation at the margins of tongue, so that fusion with superpixels was put forward to achieve the refine tongue segmentation. SpurNet fused superpixel image segmentation after the coarse segmentation of tongue features, which was used as the TCM tongue segmentation model for optimizing process of the coarse segmentation. Superpixel is defined as a set of multiple pixels with adjacent locations and similar characteristics (such as gray level and markings), which groups the pixels based on the similarity of the characteristics of different pixels. Given that SLIC [32] method is simple in thought, convenient in implementation and compact and orderly in superpixel blocks, SLIC algorithm was used by SpurNet to generate superpixels.

The SLIC algorithm procedures are as follows:

**Step 1 .**

Initialize the seed points, set the width of a image as (*N*, *N*), segmentate the image into *K* superpixels, and set the size of each superpixel as *N/K*. The step size of adjacent two seed points is:

(1) $$\begin{array}{c} S=\sqrt{\frac{N*N}{K}}. \end{array}$$

**Step 2 .**

Calculate the gradient values of all pixel points in the adjacent region *n∗n* (*n* = 3) of a seed point, and transfer the seed point to the region with the minimum gradient value to reselect a seed point.

**Step 3 .**

**Figure 5:**
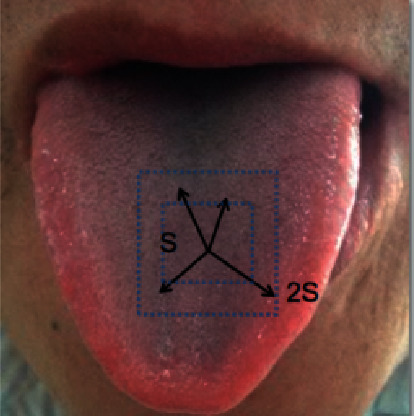
Search range of the seed points.

Calculate the distance of each pixel point and the seed point (color distance *d*_*c*_ and space distance *d*_*s*_), and allocate class tags to each pixel point in the region (*2S∗2S*) of each seed point. The search range is shown in Figure 5.

The distance measurement equation is as follow:

(2) $$\begin{array}{c} D\prime =\sqrt{{\left(\frac{d_{c}}{m}\right)}^{2}+{\left(\frac{d_{s}}{S}\right)}^{2}}. \end{array}$$

**Step 4 .**

Iterative optimization.

**Step 5 .**

Output superpixel.

**Figure 6:**
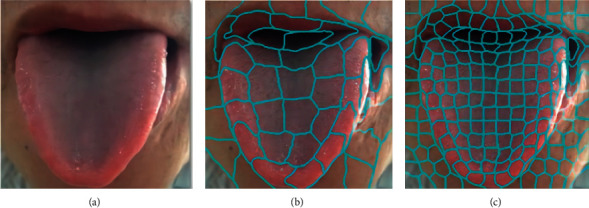
Superpixel image segmentation (a) Original image (b) Number of seed points: 50 (c) Number of seed points: 200.

When different number of seed points is selected, the superpixel image segmentation effectiveness is shown in Figure 6.

**Figure 7:**
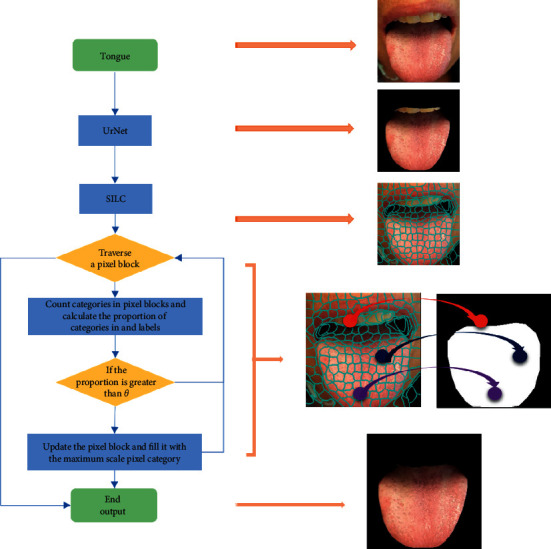
SpurNet processing procedures.

The calculation procedures of SpurNet are specified in Figure 7. Firstly, the TCM tongue images were uploaded into UrNet network, and the coarse segmentation results of tongue were obtained through procedures including convolution, downsampling, feature fusion and upsampling. Secondly, superpixel image segmentation was performed specific to the coarsely segmented images, with the number of initially selected seed points. The categories of the pixels in the same superpixel block were counted to calculate the coverage of the pixel categories in the superpixel block (relative to the segmented tongue image). For example, if the tongue pixel label was 1, the coverage of label 1 in the superpixel block was calculated. Finally, the pixel categories in each superpixel block were selectively updated, and when the coverage of tongue pixel categories was more than *θ*, the pixels in this pixel block were retained, otherwise, the image was updated as the background (label 0), so as to refine the margins of the coarsely segmented image and achieve the refined segmentation of the image.

## 3. Results

### 3.1. Dataset and Pre Processing

(1) Preprocessing: In view of the different sizes of original images and the full connection layer needed to predefine the size of weight matrix, the sizes of the images were uniformly adjusted to 256 *∗* 256*∗* 3 in advance.
To unitize the data distribution of the sample set and promote the network generalization ability, the entered images were normalized in advance and the pixel values of all samples were adjusted to [−1, +1] interval:
  (3) $$\begin{array}{c} {\text{pixel}}_{xy}=2*\left(\frac{{\text{pixel}}_{xy}}{255}\right)-1. \end{array}$$
(2) Data augmentation: Since the tongue images dataset obtained in this study was limited, data augmentation was performed before the tongue images were entered into the network, and rotatory, horizontally/vertically flipped, and translatory data augmentation was applied, with parameter settings specified in Table 1. In addition, the samples were randomly scattered before the tongue features were entered into the network in order to avoid category imbalance in the training set.

### 3.2. Experimental Settings

The model in this study was established based on Tensorflow framework. Specific training process: Initialize weight of network; upload the experimental data; extract tongue features; predict the tongue labels; update network parameters via loss calculation and back gradient propagation; use 10-fold cross-validation to assess the performance of the model.

In this study, Adam was selected as optimizer while cross-entropy as loss function, and learning rate (lr) was set as 1e-3. The learning rate attenuation strategy shown below was applied in the training process, with decay of 1e-4:

(4) $$\begin{array}{c} \text{lr}=\text{lr}*\frac{1}{1+\text{decay}*\text{iteration}}. \end{array}$$

To prevent over-fitting, the dropout was set as 0.6, and early stopping strategy was adopted in the training process. The training was stopped if the loss did not decrease on continuous 10 epochs. GPU was introduced for acceleration in the training process, with a display card of Tesla K80.

### 3.3. Contrast Test

To further verify the effectiveness of the model in the tongue segmentation, FCN and Deeplab models were used for comparison.

1. FCN. Fully convolutional network (FCN) is a semantic segmentation network proposed by Jonathan Long et al. [33] at the Institute of Electrical and Electronics Engineers (IEEE) conference in 2015. This network establishes an end-to-end and pixel-to-pixel convolutional semantic segmentation model, and is the basis of a series of semantic segmentation networks subsequently. Since FCN only contains convolutional layers without full connection layer, it can accept the input of any size, with simple structure and high efficacy.It also uses the thought of skip connection and fuses the predictive results of different depths.
2. Deeplab. Deeplab is a semantic segmentation network proposed by Chen LC and others. [34] in European Conference on Computer Vision (ECCV) in 2018 (Deeplab v3). This network applies empty convolutions with different dilation rates to capture the context information of multiscales. It establishes a simple encoder-decoder structure in which the encoder is used to capture the features of the images while the decoder to recover the specific features and spatial dimension of an image, so as to achieve the classification of pixel levels.

### 3.4. Model Assessment

Pixel accuracy (PA) [17], mean pixel accuracy (MPA) [17], mean intersection over union (MIoU) [17], and frequency weighted intersection over union (FWIoU) [17] were applied to assess the performance of the tongue feature segmentation model.

As to the same tongue image, if the label of the predicted tongue is the same as that of the artificially labeled tongue, true positive (TP) is considered, and if the predicted result is tongue while the artificially labeled result is the background, false positive (FP) is determined. Suppose the predicted result is background while the artificially labeled result is tongue, FP is considered, and if both the predicted result and the artificially labeled result are backgrounds, false negative (FN) is considered.

*Calculation equations of assessment indicators:*

PA : The proportion of the number of pixels with correctly predicted categories in the total number of pixels.

(5) $$\begin{array}{c} \text{PA}=\frac{\text{TP}+\text{TN}}{\text{TP}+\text{TN}+\text{FP}+\text{TN}}. \end{array}$$

MPA : Calculate the proportion of the number of correctly categorized pixels in each category, and then accumulate the proportions to compute the mean PA (MPA).

(6) $$\begin{array}{c} \text{MPA}=\frac{\sum_{i=1}^{n}{\text{TP}}_{i}/{\text{TP}}_{i}+{\text{FP}}_{i}}{n}, \end{array}$$

where *i* is the label *i*.

MIoU : The mean intersection over union between the predicted result by the model and the real value in each category. Since binary variables (tongue and background) were discussed in this study, the equation was:

(7) $$\begin{array}{c} \text{MIoU}=\frac{1}{2}\left(\frac{\text{TP}}{\text{TP}+\text{FP}+\text{FN}}+\frac{\text{TN}}{\text{TN}+\text{FN}+\text{FP}}\right). \end{array}$$

FWIoU : A algorithm slightly promoted on basis of MIoU, in which the weight was set based on the frequency in each category.

(8) $$\begin{array}{c} \text{FWIoU}=\frac{\text{TP}+\text{FN}}{\text{TP}+\text{FP}+\text{FN}+\text{TN}}*\frac{\text{TP}}{\text{TP}+\text{FP}+\text{FN}}. \end{array}$$

### 3.5. Experimental Results

The 10-fold cross-validation was used to train the model, and results was shown in Table 2. Comparing with FCN and DeepLab, the UrNet has a better performance on all the indicators with *PA* 0.9130 ± 0.0039, *MPA* 0.9159 ± 0.0046, *MIoU* 0.8393 ± 0.0065 and *FWIoU* 0.8430 ± 0.0065.

**Figure 8:**
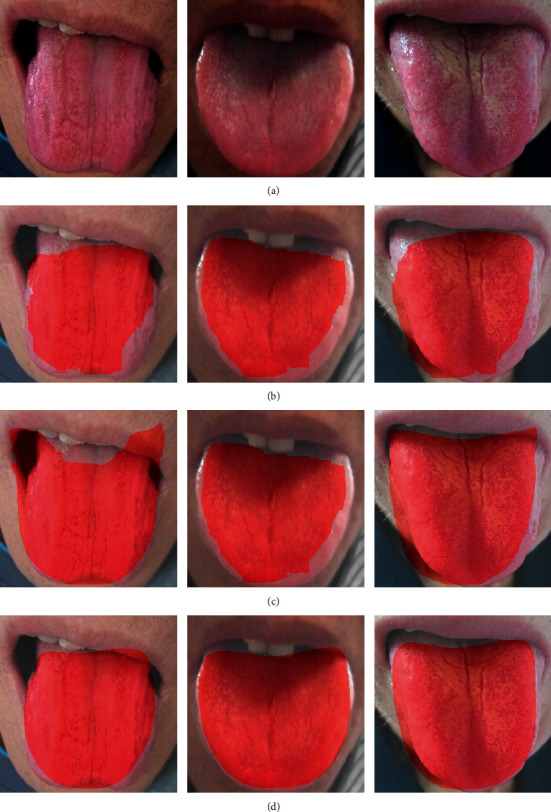
Comparison of tongue segmentation results among different models (a) Tongue (b) FCN (c) Deeplab (d) UrNet.

In order to observe the performance of the algorithm on the tongue images, the visualized segmentation results are shown in Figure 8 where the segmented tongue regions are marked red.

Based on the analysis of visualized results, the algorithms based on deep learning could approximately localize the tongue and were insensitive to the environment around the tongue, and showed intensive adaptability to the TCM tongue images. As visually shown in Figure 8, the tongue edge segmented by UrNet is smoother and the segmented area is more complete. However, after FCN and Deeplab segmentation, the margins of tongue were relatively coarse, which might cause the presence of false teeth marks at the margins of tongue, thus leading negative influence on the subsequent quantitative analysis. Therefore, UrNet adopted in this study was more advisable for tongue segmentation.

The UrNet has a better performance than FCN and DeepLab, however, when the color of lips is similar to the tongue, there are still some biases during the segmentation. To improve the performance of the UrNet in further, the SpurNet, UrNet fused with superpixel, was proposed.

**Figure 9:**
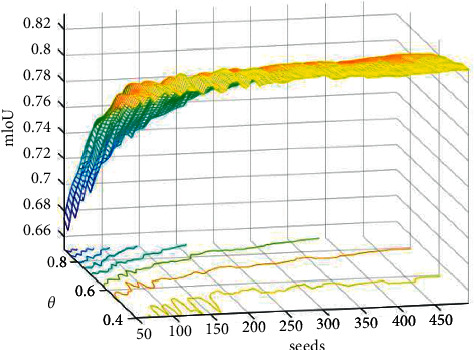
Superpixel segmentation parameter results (*X*-axis is the seeds, *Y*-axis is the *θ*, and *Z*-axis is mIoU).

In order to find the appropriate superpixel segmentation parameters, we test the number of superpixel seeds and the pixel proportion *θ* (proportion of tongue label pixels in super pixel block) in SpurNet, and the results are shown in Figure 9.

**Figure 10:**
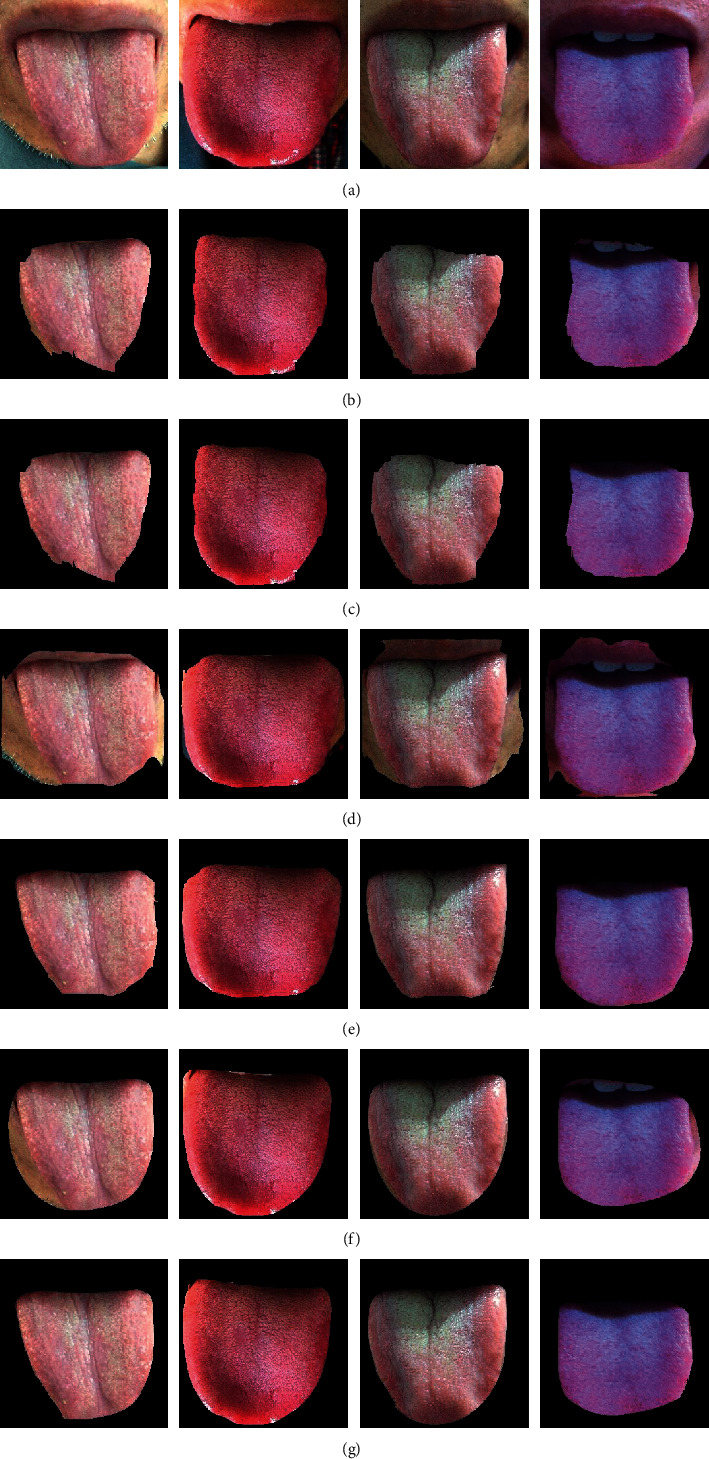
Comparison of segmentation results among different models (a) Tongue (b) FCN (c) FCN + superpixel (d) Deeplab (e) Deeplab + superpixel (f) UrNet (g) SpurNet.

In Figure 9, the mIoU tends to be stable with the increase of the number of seeds. In the subsequent experiments, we searches for the best superpixel parameters in the parameter space and fuses them with FCN, deeplab and Urnet respectively. The segmentation results of FCN, DeepLab, UrNet fused with superpixel (SpurNet) separately were compared in Table 3:

The visualized segmentation results after fusion with superpixel characteristics are shown in Figure 10.

As shown in Table 3, after superpixel characteristics were fused for post-processing, the segmentation results were promoted to certain extent than before. FCN with superpixels was superior to FCN by 0.98%, 0.71%, 1.51%, and 1.55% on indicators PA, MPA, MIoU, and FWIoU, respectively. Deeplab with superpixels was superior to Deeplab by 0.93%, 0.83%, 1.46%, and 1.47%, and SpurNet was superior to UrNet by 0.15%, 0.09%, 0.24%, and 0.24%, respectively. Relative to FCN, DeepLab and their superpixel fused models, the SpurNet model was superior in tongue image segmentation and could increase PA by 1.91%–3.17%, MPA by 1.38%–2.61%, MIoU by 3.09%–5.07%, and FWIoU by 3.11%–5.08%. Compared to UrNet, the first stage processing module, the SpurNet model also increased the PA, MPA, MIoU and FWIoU by 0.15%, 0.09%, 0.24% and 0.24%, respectively. Moreover, it was visually shown in Figure 10 that after refined segmentation by the model combined with superpixels, the contour margins were more approximate to the margins of tongue.

## 4. Discussion

Tongue diagnosis is one of the cores in TCM syndrome differentiation and has exerted a dominant function in the TCM clinical diagnosis. The waxing and waning of the healthy state can be judged and the depth of the disease location can be distinguished through tongue diagnosis to further identify the nature of the pathogenic factors and predict the medical conditions. However, the traditional TCM diagnosis of tongue features mainly depend on the experience of the clinical physicians, which may be affected by the objective or subjective environment. With the development of information technology, the objectification of TCM tongue diagnosis has become one of the hot topics in the study in TCM field. Tongue segmentation, as the basis of intellectualization of tongue diagnosis, is an indispensable step in the subsequent characteristic analysis and quantitative of TCM tongue diagnosis. However, although the traditional segmentation algorithm has a good effect, it is easily affected by light, environment, and so on, resulting in an incomplete segmentation tongue. The segmentation robustness needs to be improved. In this study, the intact and refined segmentation of the tongue was achieved using convolutional neural network concomitant with superpixel, and data augmentation technique was used, which resolved the issue of less data of tongue and ensured the robustness and generalization ability of the model.

It was known from the quantitative results in Table 2 that UrNet established in this study could exclude the influence of the locations and colors of the tongue to better refine and segmentate the tongue and background. Relative to other convolutional neural network models, UrNet convolutional network was superior to FCN and Deeplab models in segmentation effectiveness because it was more sensitive to the segmentation of the margins of tongue. Given that the UNet showed U-shaped structure and fused the high-resolution information (providing the gradient information including margins and markings, for precise localization) and low-resolution information (providing the context semantic information of each image to identify the pixel category) of the same scale in each layer, so that it could provide more accurate segmentation results. Meanwhile, it could better reserve the surface information because Resnet was selected as the Backbone. Nonetheless, FCN (which only fused the multi-layer predicted results) and Deeplab (which is only the fusion of multi-scale features) does not realize the fusion of shallow and deep information, resulting in a poor segmentation effect for details.

According to the visualized results in Figure 10, when a convolutional neural network alone was used for tongue segmentation, it led to some issues such as incomplete tongue segmentation, unsmooth margins, and the presence of additional background pixels. However, when superpixels were added for post-processing, the above issues could be effectively resolved. The superpixel blocks were obtained based on current pixel points and the features of the adjacent regions. The current pixel category has a certain association with the pixel categories in the adjacent regions. If the current pixel category is predicted wrong, it can be corrected through the pixel categories in the adjacent regions. Combining the superpixel with coarse segmentation (based on convolutional neural network) could resolve the over-segmentation and under-segmentation of a pixel at margins. It was known from the quantitative results in Table 3, after fusion with superpixel image segmentation, the four assessment indicators were all significantly promoted, and the model showed intensive adaptability to the surrounding environments, indicating that the SpurNet proposed in this study could better complete the TCM tongue feature segmentation.

Although convolutional neural network fused with superpixel image segmentation promoted the accuracy of segmentation in this study, there are still some defects urgently to be resolved: (1) The dataset labels mainly come from artificial labels, which may cause some inevitable errors. Therefore, it needs to design a procedure or depend on a clinical physician to assess whether the error range is reasonable objectively. (2) The clustering operation of super pixels is non differentiable, which makes it impossible to use back propagation for deep learning. In the follow-up work, we intend to integrate super pixels into the network to optimize the segmentation effect through network calculation. (3) This study focused on the segmentation of 2D static images and did not achieve real-time segmentation of the dynamic tongue images. Therefore, how to accurately segmentate the tongue from dynamic images and abstract and analyze the characteristics of the dynamic tongue features urgently needs to be resolved in future study. In the future, we will collect more tongue images, aiming to verify and promote the performance of tongue segmentation model in a larger and broader dataset, and try to study the subsequent models such as tongue feature extraction, classification, and so on, aiming to achieve the intellectualization of tongue diagnosis.

## 5. Conclusion

This study mainly explored the design and application of a tongue segmentation model (SpurNet) fusing convolutional neural network and superpixel characteristics, in which a tongue segmentation model containing an encoder-decoder structure was formed via introducing the residual backbone into the classic UNet model, and the segmentation results were optimized using superpixels. The experimental results have concluded that the SpurNet model, after fusing with superpixel image segmentation, is superior to classic deep learning segmentation model in segmentation effectiveness and can more accurately process the margins of tongue and resolve the over-segmentation and under-segmentation. The thought of this study is a new exploration of the deep learning in the tongue feature segmentation field, which can provide a reference for the intelligent study on tongue images.

## Figures and Tables

**Table 1:** Data augmentation parameters.

Data augmentation mode	Parameters
Rotation_range	10
Width_shift_range	0.2
Height_shift_range	0.2
Channel_shift_range	0.2
Horizontal_flip	True
Vertical_flip	True

**Table 2:** Convolutional neural network segmentation results (mean ± standard deviation).

Algorithm	*P*A	MPA	MIoU	FWIoU
FCN	0.8856 ± 0.0099	0.8959 ± 0.0079	0.7957 ± 0.0153	0.7988 ± 0.0153
DeepLab	0.8828 ± 0.0070	0.8907 ± 0.0064	0.7910 ± 0.0115	0.7946 ± 0.0110
UrNet	**0.9130** ± **0.0039**	**0.9159** ± **0.0046**	**0.8393** ± **0.0065**	**0.8430** ± **0.0065**

The best result on each metric is shown in bold face.

**Table 3:** Segmentation results after fusion with superpixel (mean ± standard deviation).

Algorithm	*P*A	MPA	MIoU	FWIoU
FCN	0.8856 ± 0.0099	0.8959 ± 0.0079	0.7957 ± 0.0153	0.7988 ± 0.0153
FCN + superpixel	0.8954 ± 0.0079	0.9030 ± 0.0070	0.8108 ± 0.0128	0.8143 ± 0.0126
DeepLab	0.8828 ± 0.0070	0.8907 ± 0.0064	0.7910 ± 0.0115	0.7946 ± 0.0110
DeepLab + superpixel	0.8921 ± 0.0049	0.8990 ± 0.0050	0.8056 ± 0.0082	0.8093 ± 0.0078
UrNet	0.9130 ± 0.0039	0.9159 ± 0.0046	0.8393 ± 0.0065	0.8430 ± 0.0065
SpurNet (UrNet + superpixel)	**0.9145** ± **0.0043**	**0.9168** ± **0.0048**	**0.8417** ± **0.0072**	**0.8454** ± **0.0072**

The best result on each metric is shown in bold face.

## Data Availability

The experimental data used to support the findings of this study are available from the corresponding author upon reasonable request (yangtao@njucm edu cn).
